# Yme2, a putative RNA recognition motif and AAA+ domain containing protein, genetically interacts with the mitochondrial protein export machinery

**DOI:** 10.1515/hsz-2021-0398

**Published:** 2022-07-26

**Authors:** Nupur Sharma, Christof Osman

**Affiliations:** Faculty of Biology, Ludwig Maximilian University Munich, D-82152 Planegg-Martinsried, Germany; Graduate School of Life Sciences, Ludwig Maximilian University Munich, D-82152 Planegg-Martinsried, Germany

**Keywords:** MBA1, MDM38, mitoribosome, OXA1, RRM, Walker motifs

## Abstract

The mitochondrial respiratory chain is composed of nuclear as well as mitochondrial-encoded subunits. A variety of factors mediate co-translational integration of mtDNA-encoded proteins into the inner membrane. In *Saccharomyces cerevisiae*, Mdm38 and Mba1 are ribosome acceptors that recruit the mitochondrial ribosome to the inner membrane, where the insertase Oxa1, facilitates membrane integration of client proteins. The protein Yme2 has previously been shown to be localized in the inner mitochondrial membrane and has been implicated in mitochondrial protein biogenesis, but its mode of action remains unclear. Here, we show that multiple copies of Yme2 assemble into a high molecular weight complex. Using a combination of bioinformatics and mutational analyses, we find that Yme2 possesses an RNA recognition motif (RRM), which faces the mitochondrial matrix and a AAA+ domain that is located in the intermembrane space. We further show that *YME2* genetically interacts with *MDM38*, *MBA1* and *OXA1*, which links the function of Yme2 to the mitochondrial protein biogenesis machinery.

## Introduction

The Mitochondrial Inner Membrane (MIM) is one of the most protein-rich membranes in the cell, as it is home to the five respiratory complexes of the electron transport chain (ETC) that are responsible for energy production by the process of oxidative phosphorylation (Acín-Pérez et al. 2008; Cogliati et al. 2018). The assembly of the ETC complexes is an intricate process that requires coordinated assembly of proteins from a bi-genomic origin. The mitochondrial DNA (mtDNA) encodes for seven subunits of the ETC (in *Saccharomyces cerevisiae)*, while the remaining subunits are encoded by the nuclear DNA (Turk et al. 2013). Mitochondria have developed sophisticated import pathways to facilitate import of nuclear DNA-encoded proteins and export of mtDNA-encoded proteins into the MIM, in order to form functional respiratory complexes (Wiedemann and Pfanner 2017). For the export of mtDNA-encoded proteins, multiple ribosome interactors, including Mdm38, Mba1 and Mrx15, have been proposed to recruit the mitoribosome to the MIM (Bauerschmitt et al. 2010; Frazier et al. 2006; Möller-Hergt et al. 2018; Ott et al. 2006). Protein insertion into the MIM then occurs co-translationally via the Oxa1 insertase (Hell et al. 2001; Szyrach et al. 2003). Despite the presence of dedicated and efficient import machineries, a number of protein quality control pathways exist that remove misfolded and superfluous proteins to prevent faulty complex assembly (Böttinger and Becker 2012; Tatsuta 2009). An inefficient complex assembly may lead to ROS production and have deleterious effects on mitochondrial integrity, which in turn can cause various muscular and neurodegenerative disorders in humans (Nunnari and Suomalainen 2012).

A pivotal role in mitochondrial protein quality control, is played by members of the AAA+ (ATPases Associated with diverse cellular Activities) protein family, that perform ATP-driven unfolding, extraction and degradation of damaged and dysfunctional proteins (Gates and Martin 2020; Song et al. 2021; Steele and Glynn 2019). The AAA+ proteins are hetero- or homo-oligomeric complexes, that assemble to form ring-like structures (Gerdes et al. 2012; Miller and Enemark 2016; Opalińska and Jańska 2018). AAA+ proteins belong to the superfamily of P-loop nucleoside triphosphate binding proteins (Snider et al. 2008). The hallmark of this superfamily are the ATP binding and hydrolysis domains, namely the Walker A and B motifs, respectively (Walker et al. 1982). The Walker A motif (G-x(4)-GK-[TS]) is a conserved P-loop (Phosphate loop) motif that lines the axial channel of the ring assembly and co-ordinates the beta and gamma phosphates of the nucleotide during ATP hydrolysis (Gates and Martin 2020; Wendler et al. 2012). The Walker B motif (hhhhDE, ‘h’ denoting a hydrophobic amino acid), on the other hand, contributes to ATP hydrolysis by coordinating the water-activating magnesium ion with the help of acidic residues (Hanson and Whiteheart 2005). Within the P-loop superfamily, AAA+ proteins belong to the ASCE (Additional Strand Catalytic “E”) class that is characterized by an additional β-strand that separates the P-loop and the Walker B strands in a central five-strand containing β-sheet of an α–β–α sandwich (Erzberger and Berger 2006; Seraphim and Houry 2020). A feature that sets AAA+ proteins apart from other members of the ASCE class is the absence of additional β-strands adjacent to the central β-sheet (Erzberger and Berger 2006; Miller and Enemark 2016; Puchades et al. 2020).

Mitochondrial protein quality control depends on a network of AAA+ proteins. Two important complexes are the i-AAA and the m-AAA protease, which are AAA-proteases that function to degrade misfolded proteins from the IMS and the matrix, respectively (Arlt et al. 1996; Glynn 2017; Leonhard et al. 1996; Levytskyy et al. 2017). Additionally, the AAA+ proteins Msp1 and Cdc48, are involved in extraction of proteins from translocation pores of the outer membrane (Basch et al. 2020; Mårtensson et al. 2019; Song et al. 2021; Weidberg and Amon 2018).

**Figure 1:**
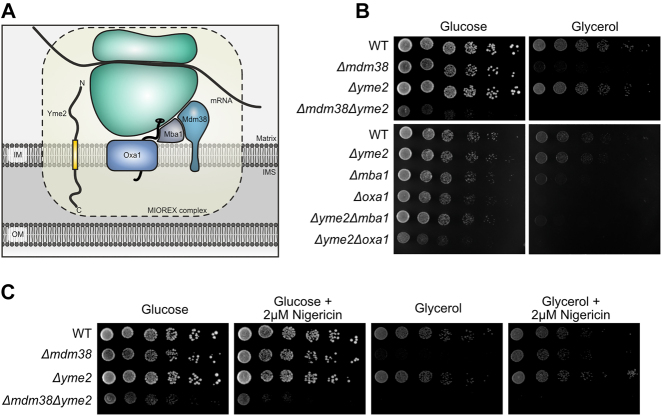
Genetic interactions of *YME2* with components of the protein biogenesis machinery. (A) A schematic showing the presence of Yme2 in the MIOREX complex (Kehrein et al. 2015), consisting of the mitochondrial ribosome and its interactome. Yme2, a MIM protein of 96 kDa, has been proposed to have an N-terminal matrix facing domain (32 kDa) and a C-terminal domain (60 kDa) facing the IMS. (B) Growth test analysis showing the genetic interactions of *YME2* with *MDM38*, *MBA1*, and *OXA1*. The indicated strains were grown to logarithmic phase and the serial dilutions were spotted on fermentable glucose medium and non-fermentable glycerol medium and incubated at 30 °C for 2 days (in case of *∆mdm38, ∆yme2∆mdm38*) and 1 day (in case of *∆mba1*, *∆oxa1*, *∆yme2∆mba1*, *∆yme2∆oxa1*). (C) Growth test analysis of the indicated strains showing serially diluted cells spotted on glucose medium and glycerol medium without and with 2 µM Nigericin. The cells were incubated at 30 °C for 2 days.

*YME2* (Yeast Mitochondrial Escape protein 2) was first discovered in a genetic screen conducted in *S.cerevisiae*, wherein the loss of *YME2* led to escape of mitochondrial DNA from mitochondria to the nucleus (Hanekamp and Thorsness 1996; Thorsness and Fox 1993). Yme2 is a single-spanning trans-membrane protein of the inner mitochondrial membrane, which exposes its N- and C-termini to the matrix and intermembrane space, respectively (Figure 1A) (Hanekamp and Thorsness 1996; Leonhard et al. 2000). More recently, Yme2 was found to co-localize with mtDNA nucleoids (Murley et al. 2013) and to be associated with the MIOREX complexes, which are large expressosome-like assemblies comprising factors that are bound to mitoribosomes and are involved in mitochondrial gene expression (Kehrein et al. 2015). Here, we find that *YME2* exhibits negative genetic interactions with *MDM38*, *MBA1* and OXA1, which links *YME2* to mitochondrial protein biogenesis. Furthermore, we analyze the domain organization of Yme2 and thereby provide insights into the possible function of Yme2.

## Results

### *YME2* displays a negative genetic interaction with components of the mitochondrial protein export machinery

The presence of Yme2 in the MIOREX complex led us to examine potential genetic interactions of *YME2* with components required for mitochondrial protein biogenesis. First, we focused on a negative genetic interaction between *YME2* and *MDM38*, which has been reported previously in a systematic large scale screen (Usaj et al. 2017). Mdm38 is a protein of the inner mitochondrial membrane that acts as a receptor to recruit the mitochondrial ribosome to the MIM (Bauerschmitt et al. 2010; Frazier et al. 2006). Moreover, Mdm38 has been proposed to play a role in mitochondrial K^+^/H^+^ homeostasis (Nowikovsky et al. 2004). We generated *∆yme2*, *∆mdm38* and *∆yme2∆mdm38* strains and performed growth test analyses. In agreement with previous findings, deletion of *MDM38* caused a growth defect on non-fermentable medium, while deletion of *YME2* did not result in obvious growth defects on either fermentable or non-fermentable medium (Frazier et al. 2006; Hanekamp and Thorsness 1996). In contrast, *Δyme2Δmdm38* cells exhibited a strong growth defect even on fermentable medium and were incapable of respiratory growth at 30 and 37 °C (Figures 1B and S1). To examine whether the *Δyme2Δmdm38* phenotype was caused due to a defect in ion transport or ribosome binding, a growth test analysis was performed on media containing Nigericin. Nigericin, a K^+^/H^+^ ionophore, was previously shown to rescue the phenotype of *Δmdm38* (Nowikovsky et al. 2007). Conforming to this analysis, we observed that growth of *∆mdm38* cells is restored on non-fermentable medium in the presence of Nigericin (Figure 1C). In comparison, the severe phenotype of *Δyme2Δmdm38* cells could not be rescued by Nigericin. This result suggests that the strong negative genetic interaction between *YME2* and *MDM38* is rather linked to Mdm38’s function as a ribosome receptor than its role in K^+^/H^+^ homeostasis.

Mdm38 has been linked to Oxa1-mediated insertion of mtDNA-encoded proteins and has also been reported to physically interact with Mba1 (Bauerschmitt et al. 2010). In this respect, we examined whether *YME2* also genetically interacts with *MBA1* and/or *OXA1*. Indeed, we observed slightly reduced growth of *Δyme2Δmba1* cells on non-fermentable medium and of *Δyme2Δoxa1* cells on fermentable media at 30 and 37 °C compared to the respective single mutants (Figures 1B and S1). Thus, *YME2* exhibits a negative genetic interaction with proteins of the mitochondrial export machinery and is therefore not only linked to protein biogenesis through its association with the MIOREX complex (Kehrein et al. 2015), but also through its genetic interactions. Of note, the strongest negative genetic interaction is observed between *YME2* and *MDM38*.

### Yme2 contains putative Walker motifs that are important for Yme2 function

Next, we used bioinformatics tools to obtain insight into a possible function of Yme2. Prediction of the putative structure of Yme2 was made using AlphaFold (Jumper et al. 2021). The resulting model revealed distinct N-terminal (average per residue confidence score, or pLDDT > 90) and C-terminal (average pLDDT > 70) domains that are separated by an alpha helix that corresponds to a predicted transmembrane domain between residues 287 and 305 (average pLDDT > 70) (Figure S2A).

**Figure 2:**
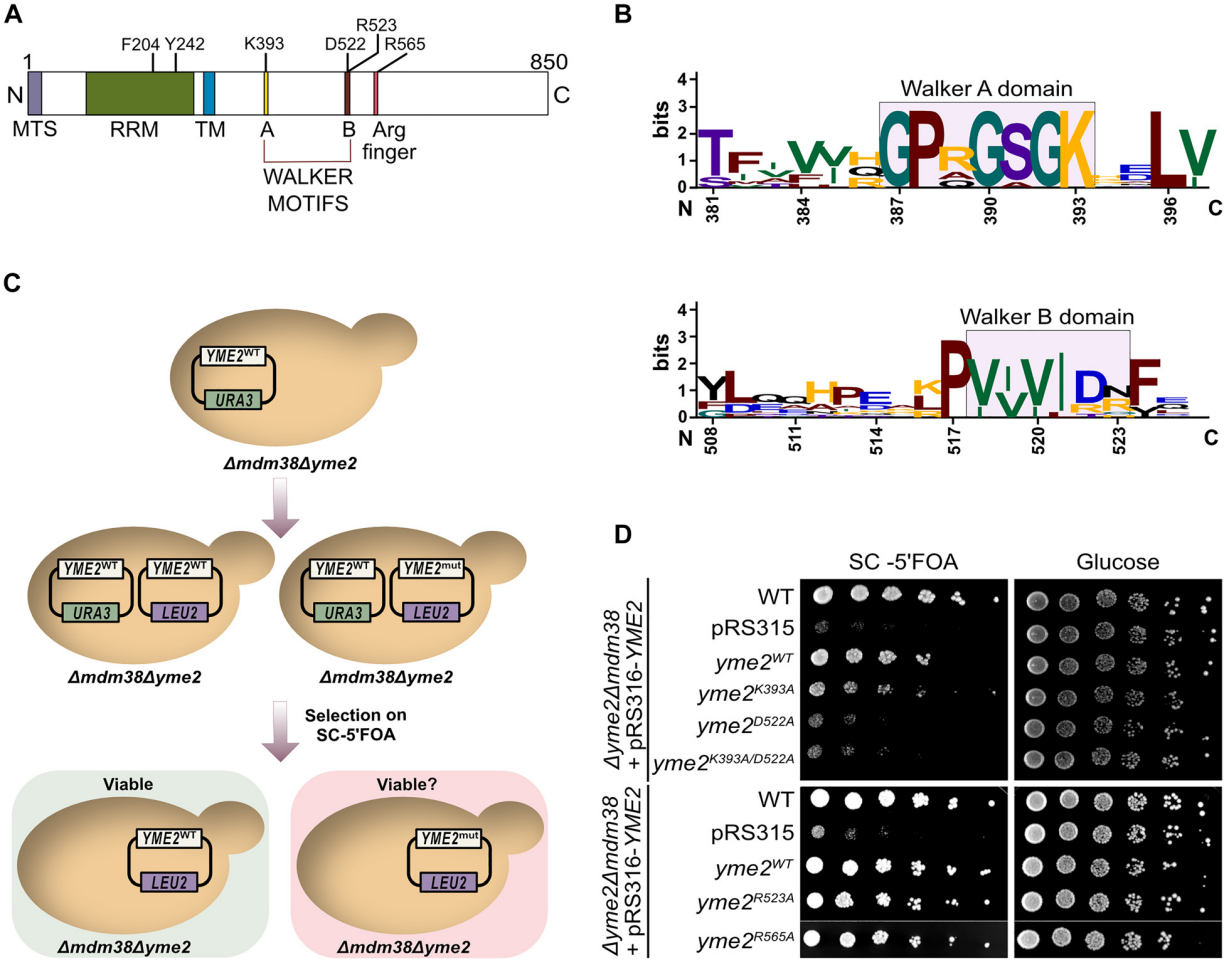
Yme2 contains a putative AAA+ domain. (A) Schematic showing the predicted domain organization of Yme2. The sequence of Yme2 harbours a mitochondrial targeting signal followed by a putative N-terminal RNA binding domain (RRM), a putative C-terminal AAA+ domain with the predicted Walker A and B motifs, separated by a predicted transmembrane (TM) domain. It also possesses an additional putative Arginine finger residue. The indicated residues were mutated in this study. (B) Sequence logo generated from https://weblogo.berkeley.edu/ depicting the conservation of the sequence of Walker A and B motifs of the putative AAA+ domain of Yme2 across 10 different fungal species. The numbers marking the residues refer to the Yme2 sequence in *S. cerevisiae*. (C) Schematic of the experimental procedure for the plasmid shuffle experiment. (D) Growth test analysis showing the plasmid shuffle experiment. The indicated strains were grown to logarithmic phase and the serial dilutions were spotted on fermentable glucose medium and on SC+ 5 FOA medium (to counter select for the pRS316-*URA3* plasmid) and incubated at 30 °C for 2 days.

A careful analysis of the Yme2 amino acid sequence using a homology search based on 3D structure prediction (Meier and Söding 2015; Söding et al. 2005; Zimmermann et al. 2018), revealed similarities of the Yme2’s IMS domain with AAA+ proteins and the presence of putative Walker A and B motifs characteristic of P-loop ATPases (Figure 2A) (Miller and Enemark 2016; Puchades et al. 2020). The strongest similarities in the homology search were identified among members of the DNA-binding initiator clade of AAA+ proteins, which include origin recognition proteins and helicase-loading proteins, such as Cdc6. This finding is rather surprising given the localization of this domain of Yme2 in the IMS, which supposedly lacks DNA or RNA. Inspection of the predicted AlphaFold structure of Yme2’s IMS domain supported the presence of a AAA+ fold, because it revealed features characteristic of AAA+ proteins, which include a central β-sheet consisting of five β-strands with a β5–β1–β4–β3–β2 order, a second region of homology connecting β4 and β5 (including a hydrophilic glutamate at position 558 that may serve as a putative Sensor I and a putative Arginine finger at position 565) and a helical bundle C-terminal to the α–β–α sandwich (Figure S2B) (Miller and Enemark 2016; Puchades et al. 2020). The Walker A motif is typically found in a loop connecting β1 and β2 and contains an invariant lysine residue which, when mutated, has been observed to abolish ATP binding (Wendler et al. 2012). In case of Yme2 in *S. cerevisiae*, this lysine is at position 393 (Figures 2B and S2C). The Walker B motif that typically contains two conserved acidic residues (commonly an Aspartate and Glutamate), has an unusual replacement of the Glutamate with an Arginine residue in the Yme2 sequence (Figure 2B and S2C) (consensus: hhhhDE; Yme2: hhhhDR) (Hanson and Whiteheart 2005; Wendler et al. 2012). To examine the importance of the Walker A and B motifs, the unusual Arginine in the Walker B motif and the putative Arginine finger for Yme2 function, we generated *YME2* variants with Walker A (*yme2*^*K393A*^), Walker B (*yme2*^*D522A*^), Walker A/B (*yme2*^*K393A/D522A*^), Walker B-Arginine (*yme2*^*R523A*^) and Arginine finger (*yme2*^*R565A*^) mutations and assessed their ability to rescue the *Δyme2Δmdm38* growth phenotype in a plasmid shuffle experiment (Figure 2C). *Δyme2Δmdm38* cells expressing *YME2* from a centromeric plasmid containing a *URA3* marker were transformed with a plasmid containing the *LEU2* marker, which either harbored the WT or mutated forms of *YME2*. The cells were subsequently grown on SC medium supplemented with 5′FOA, which only allows growth of cells that have lost the URA3-marked plasmid. In cells that have retained the URA3 plasmid, 5′FOA gets converted to a toxic product by the Ura3 protein, which kills the cell. In this assay, we observed that the *yme2*^*R523A*^ and *yme2*^*R565A*^ variants containing the mutations of the Walker B-Arginine or the putative Arginine finger, respectively, rescued growth of *Δyme2Δmdm38*, indicating that these residues are not essential in the absence of Mdm38. Most interestingly, the Walker mutant variants *yme2*^*K393A*^, *yme2*^*D522A*^ and *yme2*^*K393A/D522A*^ failed to efficiently rescue the growth defect associated with *Δyme2Δmdm38* cells (Figures 2D and S2D). Of note, we observed a weak rescuing effect of the *yme2*^*K393A*^ variant, indicating that this mutation does not entirely abolish Yme2 function. To test if all mutant Yme2 forms are expressed and to avoid that the strong *Δyme2Δmdm38* phenotype may affect protein levels, we transformed *Δyme2* cells with plasmids encoding mutant variants and checked expression levels in cell lysates by Western bloting (Figure S2E). These analyses revealed that all mutant forms are expressed to levels comparable to wildtype Yme2. Taken together, we conclude that the Walker motifs are critical for Yme2 function.

### Yme2 forms high molecular weight complexes

**Figure 3:**
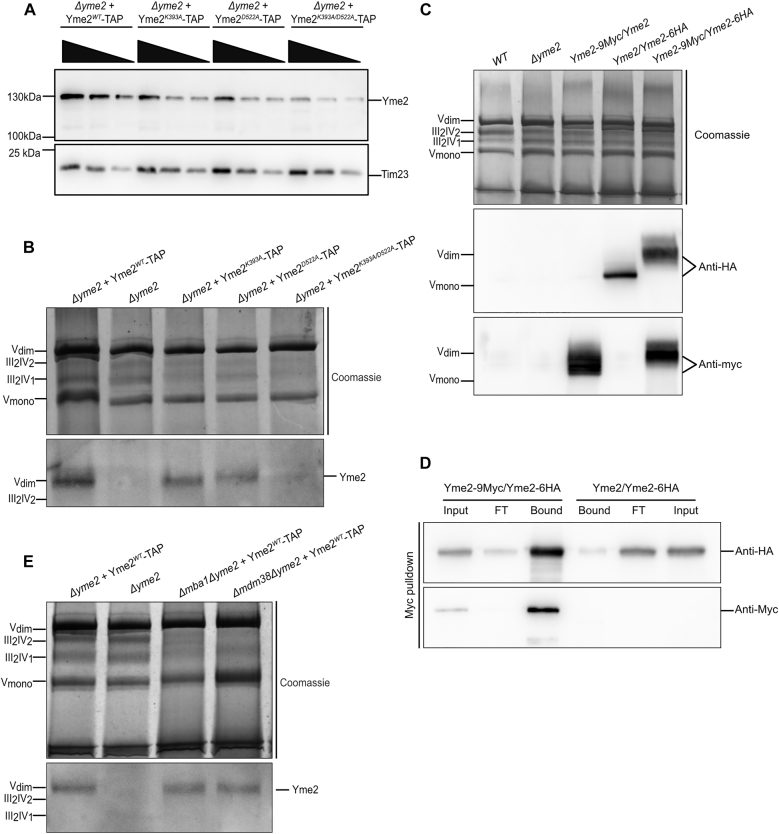
Yme2 forms high molecular weight complexes. (A) Western blot of the isolated mitochondria of the respective strains showing the steady state protein levels upon immunodetection with the Yme2-antibody. For each strain, 50, 25 and 12.5 µg mitochondria were loaded on the SDS-PAGE. Tim23 is the loading control. (B, C) Western Blot of the isolated mitochondria loaded on a Blue-Native PAGE. For each sample, 100 µg of mitochondria were solubilized and loaded on a 3–13% BN-PAGE. The western blot was probed with the indicated antibodies. The Coomassie staining shows the mitochondrial respiratory supercomplexes, as indicated. (D) Western blot showing the Myc-immunoprecipitation experiment performed with the indicated diploid strains. For each strain, 1% of the input and flowthrough (FT), and 50% of the bound fractions were loaded. The blot was decorated with Anti-HA and Anti-Myc antibody. (E) Western Blot of the isolated mitochondria from the indicated strains loaded on a Blue-Native PAGE. For each sample, 100 µg of mitochondria were solubilized and loaded on a 3–13% BN-PAGE. The western blot was probed with an anti-Yme2 antibody. The Coomassie staining shows the mitochondrial respiratory supercomplexes, as indicated. The deletion strains *∆mba1* and *∆mdm38* show reduced levels of supercomplexes, due to an absence of the components of the mitochondrial protein biogenesis machinery.

Given the predicted AAA+ fold of Yme2 and the importance of the Walker motifs for Yme2 function, we asked whether Yme2 forms oligomeric complexes, which is a characteristic feature of AAA+ proteins (Puchades et al. 2020). To facilitate detection of Yme2 in BN-PAGE analysis, WT or mutant Walker A or B TAP-tagged *YME2* variants were re-inserted into the *LEU2* locus of a *Δyme2* strain. Importantly, the TAP-tag did not interfere with Yme2 function, which was evident by WT-like growth of a *∆mdm38 YME2-TAP* strain (Figure S3A). The steady state protein levels of all Yme2 variants were examined in isolated mitochondria (Figure 3A). We observed that strains expressing TAP-tagged WT Yme2, Yme2^K393A^ or Yme2^D522A^ displayed comparable Yme2 protein levels, indicating that these individual mutations do not cause protein instability, similar to our observation of untagged Yme2. In contrast, however, Yme2^K393A/D522A^-TAP levels were slightly lower. We interpret this decrease in protein levels of Yme2^K393A/D522A^-TAP to reflect a slight destabilization of the protein, which becomes apparent upon isolation of mitochondria. Blue Native PAGE and Western Blot analysis of mitochondria isolated from strains expressing TAP-tagged Yme2 revealed a sharp Yme2-specific band, which ran at a high molecular weight size similar to dimeric complex V (∼1250 kDa) (Figure 3B). This Yme2-complex was also apparent in mitochondria isolated from cells expressing the Walker A mutant form Yme2^K393A^. In contrast, complex formation of Yme2 was compromised in the presence of the Walker B mutation and virtually absent in cells expressing the Yme2^K393A/D522A^ double mutant form harbouring Walker A and B mutations.

Next we asked, whether the Yme2-complex contains multiple copies of the Yme2 protein. We generated three diploid strains containing either a 9Myc- and an 6HA-tagged *YME2* allele (Yme2-9Myc/Yme2-6HA), an untagged and an HA-tagged *YME2* allele (Yme2/Yme2-6HA), or an untagged and a Myc-tagged *YME2* allele (Yme2/Yme2-9Myc). Yme2 complexes in these strains were analysed by BN PAGE. Interestingly, the size of the Yme2 complex displayed a clear shift in Yme2-9Myc/Yme2-6HA mitochondria compared to the strains where only one allele was tagged (Figure 3C). This size shift most likely indicates that both Yme2 variants are present in the same complex. To test this further, isolated mitochondria from Yme2-9Myc/Yme2-6HA and Yme2/Yme2-6HA strains were subjected to Myc-immunopurification. Strikingly, Yme2-6HA efficiently co-purified with Yme2-9Myc in this experiment, while it did not purify when Yme2/Yme2-6HA mitochondria were subjected to Myc-purification (Figure 3D). Similarly, Yme2-9Myc could be successfully co-purified with Yme2-6HA (Figure S3B). Taken together, these results suggest that Yme2 forms a high molecular weight complex that contains multiple copies of Yme2. Furthermore, replacement of the lysine residue in the Walker A motif does not interfere with Yme2 complex formation, while mutation of the aspartate within the Walker B motif partially impairs complex formation. Mutation of Walker A and Walker B residues strongly compromises complex formation.

Given the genetic link between *YME2* and components of the mitochondrial protein export machinery, we also assessed Yme2-TAP complex formation in *∆mdm38* and *∆mba1* strains (Figure 3E). No alteration of the Yme2 complex could be observed in the absence of Mdm38 or Mba1 indicating that the Yme2 complex revealed in the BN-PAGE analysis does not contain either of these proteins and absence of these proteins does not compromise complex formation through secondary effects.

### Yme2 has a putative N-terminal RNA binding domain

**Figure 4:**
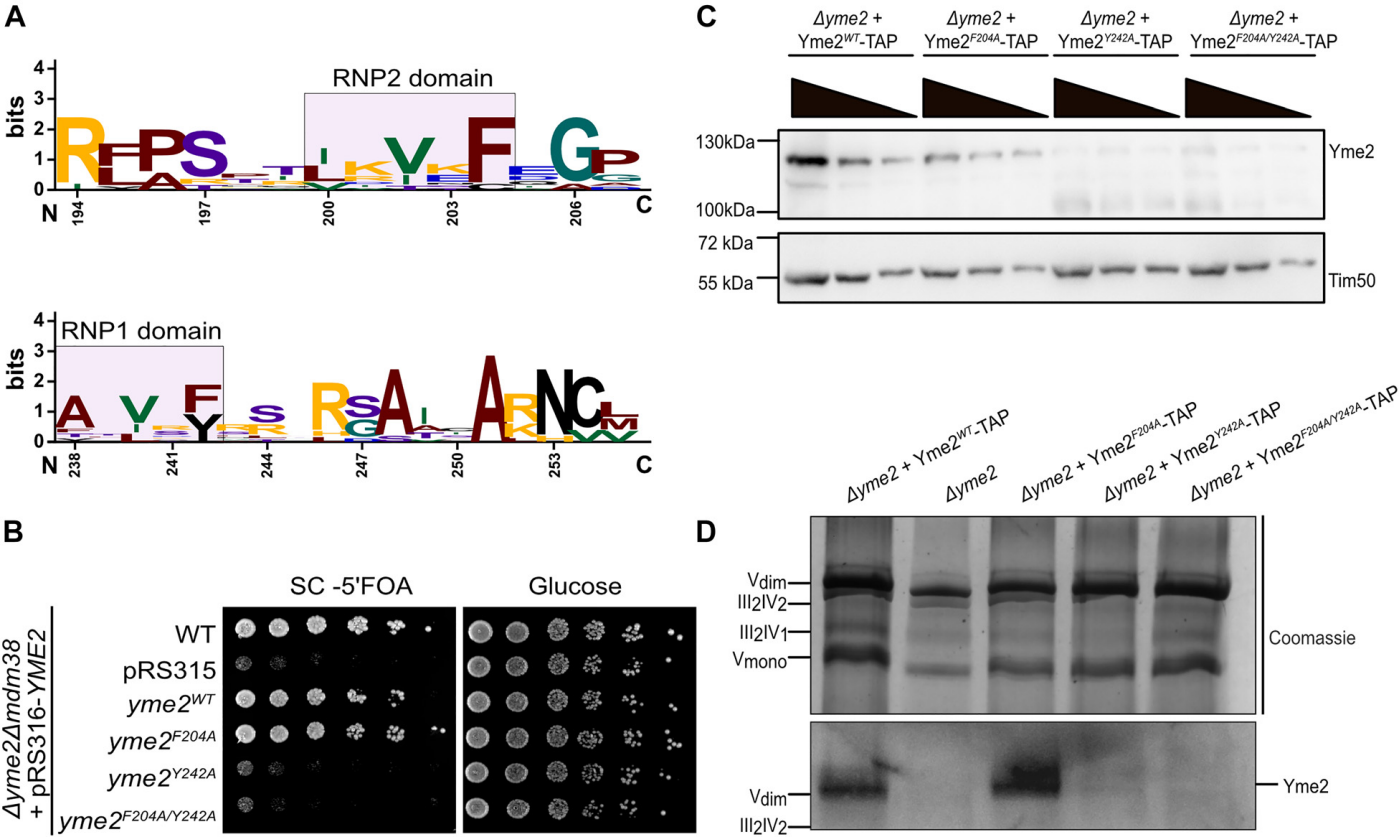
Yme2 contains a putative RRM domain. (A) Sequence logo generated from https://weblogo.berkeley.edu/ depicting the conservation of the sequence of RNP2 and RNP1 domains of the putative RRM domain of Yme2 across 10 different fungal species. The numbers marking the residues refer to the Yme2 sequence in *S. cerevisiae.* (B) Growth test analysis showing the plasmid shuffle experiment performed with the RNP mutant forms of *YME2*. The indicated strains were grown to logarithmic phase and the serial dilutions were spotted on fermentable glucose medium and on SC+ 5 FOA medium (to counter select for the pRS316-*Ura3* plasmid) and incubated at 30 °C for 2 days. (C) Western Blot of the isolated mitochondria of the respective strains showing the steady state protein levels upon decoration with the Yme2-antibody. For each strain, 50, 25 and 12.5 µg mitochondria were loaded on the SDS-PAGE. Tim50 is the loading control. (D) Western Blot of the isolated mitochondria loaded on a Blue-Native PAGE. For each sample, 100 µg of mitochondria were solubilized and loaded on a 3–13% BN-PAGE. The western blot was decorated with an antibody against Yme2. The Coomassie staining shows the mitochondrial respiratory supercomplexes, as indicated.

We next focused on the domain of Yme2, which is located in the mitochondrial matrix. Analysis of the amino acid sequence revealed the presence of a putative N-terminal RNA binding domain (Gabler et al. 2020; Meier and Söding 2015; Nowacka et al. 2019; Söding et al. 2005; Zimmermann et al. 2018). The RNA recognition motif (RRM) domain has two conserved motifs, namely RNP1 and RNP2, wherein each motif contains an invariant aromatic residue that interacts with the nucleotide bases of a client RNA or DNA molecule (Figures 4A and S4A) (Maris et al. 2005). The RNP motifs of Yme2 were observed to be conserved among various fungal species (Figure S4B). To test whether motifs are important for the function of Yme2, we examined if mutant *YME2* variants containing RNP1 (*yme2*^*Y242A*^) and RNP2 (*yme2*^*F204A*^) mutations would rescue growth of a *Δyme2Δmdm38* strain in our plasmid shuffle experiment (Figure 2C). In this assay, we observed that the RNP2 mutation (*yme2*^*F204A*^) did not interfere with Yme2 function because a plasmid containing this variant restored growth of *Δyme2Δmdm38* cells (Figures 4B and S4C). Thus, mutation of the phenylalanine does not impair the function of the RNP2 motif. In contrast, the RNP1 mutation (*yme2*^*Y242A*^) or the RNP1/RNP2 double mutation (*yme2*^*F204A/Y242A*^) rendered Yme2 non-functional in the plasmid shuffle experiment.

We next determined the stability of Yme2 variants containing RRM mutations and transformed *Δyme2* cells with plasmids encoding either WT or mutant forms of *YME2*. While the *Yme2*^*F204A*^ variant accumulated to WT levels, amounts of *Yme2*^*Y242A*^ and *Yme2*^*F204A/Y242A*^ were not detectable in cell lysates (Figure S4D). We also examined levels of Yme2 variants containing mutations of the RRM domain in mitochondria isolated from strains containing TAP tagged versions integrated into the *LEU2* locus. In agreement with our results obtained from cell lysates, the *Yme2*^*F204A*^-TAP levels were comparable to WT levels (Figure 4C). Yme2^Y242A^-TAP and Yme2^F204A/Y242A^-TAP levels, in contrast, were strongly reduced and only residual amounts were detectable. To examine complex formation of Yme2 variants containing RRM mutations, we performed BN-PAGE analysis. In line with our findings that mutation of the RNP2 motif does not interfere with the function of Yme2 and does not affect its abundance, we observed that the *Yme2*^*F204A*^ variant formed WT-like high molecular weight complexes. In contrast, no complexes were observed for the *Yme2*^*Y242A*^ and *Yme2*^*F204A/Y242A*^ mutants (Figure 4D). We conclude that the RNP1 motif is essential for Yme2 stability and complex formation. However, it is indistinguishable at this point, whether the integrity of RNP1 is required for folding of Yme2 or whether RNA binding plays an important role in stabilizing or promoting complex formation and protein stability.

## Discussion

In this study, we demonstrate that *YME2* negatively interacts with *MDM38*, *MBA1* and *OXA1*, which all have proposed roles in mitochondrial protein biogenesis. The strongest negative genetic interaction of *YME2* is apparent in *∆yme2∆mdm38* cells, where a severe growth defect is observed even on fermentable medium, which cannot be rescued by the addition of Nigericin. These results suggest a partially overlapping role of Yme2 and Mdm38, which is crucial for mitochondrial and cellular function. Our bioinformatic and mutational analyses shed light on the domain organization of Yme2. Yme2 is predicted to possess a putative RNA Recognition Motif (RRM), that faces the mitochondrial matrix and an AAA+ domain, that faces the IMS. Based on our mutational analyses, both domains are important for Yme2 function.

The genetic interaction of Yme2 with components of the mitochondrial export machinery combined with the previous observation that Yme2 co-purifies with the mitochondrial ribosome (Kehrein et al. 2015) suggests a function for Yme2 in mitochondrial protein biogenesis. Yme2’s RRM domain would be well-positioned in the mitochondrial matrix to assist in this process. RRM domains are structurally versatile and have been shown to engage in RNA, DNA or protein interactions (Maris et al. 2005). Therefore, it remains to be determined which of these molecules Yme2 interacts with through its RRM domain. Given the strong genetic interaction between *YME2* and *MDM38* and the proposed function of Mdm38 as a ribosome acceptor at the MIM (Frazier et al. 2006), an interesting hypothesis would be that Yme2 assists in protein translation by binding to either mRNAs or rRNAs through its RRM domain to tether and position components of the translation machinery at the MIM. According to this idea, Yme2 and Mdm38, may serve partially redundant roles in tethering the translation apparatus to the MIM to facilitate efficient protein insertion. In absence of either *YME2* or *MDM38*, the respective other protein would still be sufficient for this function. In *∆yme2∆mdm38* cells, however, tethering and precise positioning of the mitoribosome could be compromised to an extent that severely hampers protein insertion and results in the observed growth defects. An alternative hypothesis could be that Yme2 engages in DNA interactions through its RRM domain and facilitate mtDNA recruitment to the MIM, which could spatially link transcription and translation. Such a function would be in accordance with the previously observed co-localization of Yme2 and mtDNA (Murley et al. 2013).

It has to be pointed out that the strong growth defect of *∆yme2∆mdm38* cells even on fermentable medium is surprising, if the function of Yme2 and/or Mdm38 lie solely in the membrane integration of mtDNA-encoded proteins. It will be interesting to examine potential functions of Yme2 and Mdm38 beyond export of mtDNA-encoded proteins.

The AlphaFold-based structural prediction suggests that Yme2 adopts a AAA+ fold in the IMS and our results demonstrate that the Walker motifs are indispensable for Yme2’s function. Furthermore, the presence of Yme2 in a high molecular weight complex containing multiple copies of Yme2 is reminiscent of the known homo- or hetero-oligomeric organizations of AAA+ proteins (Miller and Enemark 2016). However, the Walker B motif of Yme2 (hhhhDR) differs from the consensus sequence of the Walker B motif (hhhhDE) (Hanson and Whiteheart 2005) and mutation of the putative Arginine finger (Arg 565) appears dispensable for Yme2 function. These points raise the question if Yme2 indeed has the ability to hydrolyze nucleoside triphosphates and to function as a motor in the IMS akin to other AAA+ proteins. In any case, identification of molecules that interact with its AAA+ domain will be critical to unravel the function of Yme2.

In summary, our analyses link Yme2 to the mitochondrial protein biogenesis machinery. The identification of the RRM and AAA+ domains within Yme2 further provide a strong foothold into mechanistic analyses of Yme2’s function.

## Materials and methods

### Construction of yeast strains and plasmids

All the yeast strains used in the study were generated in the W303 background. Single deletion mutants and strains with C-terminally tagged genes were constructed as previously described (Janke et al. 2004). Double deletion mutant strains were constructed by mating the respective single deletions followed by tetrad dissection analysis. The list of all yeast strains used is given in Supplementary Table S1. The strain used for the plasmid shuffle was constructed by first deleting *YME2* in WT background, followed by transformation with pRS316*-YME2*. This strain was further used for deletion of *MDM38*. The plasmid shuffle experiment was performed with centromeric yeast vectors pRS315 and pRS316. All the plasmids used in the study are listed in Supplementary Table S2. For the SDS-PAGE and BN-PAGE analysis, TAP-tagged *YME2* strains were generated, by integration of WT or mutated forms of *YME2-TAP* into the *LEU2* locus of a *Δyme2* strain. Primer sequences can be made available on request.

### Growth test analysis

Growth test analysis was performed using log-phase growing cells in YPD, and taking equal number of cells for the respective strains followed by spotting them on fermentable (YPD) and non-fermentable (YPG) media, at 30 and 37 °C. Pictures were taken after 24 and 48 h of growth at the respective temperatures. For the plasmid shuffle, the cells were additionally spotted on SC medium supplemented with 5′-fluoroorotic acid (FOA), to select for cells that have lost the pRS316-*URA3* plasmid.

### Mitochondria isolation

The protocol for isolating the mitochondria was adapted from (Basch et al. 2020). The final mitochondrial pellet was resuspended in SEM buffer (250 mM Sucrose, 1 mM EDTA, 10 mM MOPS-KOH pH 7.2), and further subjected to sucrose step gradient centrifugation over SEM_500_ buffer (500 mM sucrose, 1 mM EDTA, 10 mM MOPS-KOH pH 7.2) at 13,000 rpm for 10 min (Morgenstern et al. 2017). The pellet was further resuspended in SEM buffer, aliquoted and shock frozen in liquid N_2_.

### Immunoprecipitation experiments

Mitochondria were lysed in 2% Digitonin, 50 mM NaCl, 10 mM Tris-HCl (pH 7.4) and 1x Complete protease inhibitor (Roche) for 30 min at 4 °C. The lysate was cleared by centrifugation at 13,000*g* for 10 min at 4 °C. The lysate was incubated for 2 h with Anti-c-Myc magnetic beads (Pierce™) or Anti-HA beads (Pierce™) at 4 °C. Bound proteins were eluted with Laemmli buffer and analysed by Western blotting.

### PAGE analysis

For the SDS-PAGE, 50, 25 and 12.5 µg were centrifuged at 13,000 rpm for 10 min at 4 °C, and the pellet was resuspended in Laemmli buffer, and analyzed by SDS-PAGE and immunoblotting.

The protocol for BN-PAGE was adapted from (Wittig et al. 2006). 100 µg mitochondria were pelleted at 13,000 rpm for 10 min at 4 °C. The pellet was resuspended in solubilization buffer (50 mM NaCl, 5 mM 6-aminohexanoic acid, 50 mM imidazole/HCl pH 7.0, 50 mM K_2_PO_4_ pH 7.4, 10% (v/v) glycerol) with 1.875% digitonin, and incubated for 20 min at 4 °C. Subsequently, samples were centrifuged at 20,000*g* for 20 min at 4 °C. The supernatant was mixed with 2 µL solubilization buffer containing 2% Coomassie Brilliant Blue G-250, loaded on a 3–13% BN-PAGE, and analyzed by immunoblotting.

### Multiple sequence alignment and structure prediction

Yme2 sequence from 10 different fungal species was aligned using MUSCLE (Madeira et al. 2019), and analyzed using Jalview (Waterhouse et al. 2009). The sequence logo for the Walker A and B motifs, and the RNP 1 and 2 motifs was further created using the Weblogo tool (Crooks et al. 2004). For the prediction of the structure of Yme2, the AI-based system AlphaFold (Jumper et al. 2021) was used.

## Supplementary Material

Supplementary Material
