# Supplementary material for: Yme2, a putative RNA recognition motif and AAA+ domain containing protein, genetically interacts with the mitochondrial protein export machinery
Source: Biol Chem. 2022 Jan 31;403(8-9):807–17. doi: 10.1515/hsz-2021-0398 (PMC9284673; doi:10.1515/hsz-2021-0398)
Supplement: Supplementary file 1 — Supplementary Material [file j_hsz-2021-0398_suppl.pdf]

Yme2, a putative RNA Recognition motif and AAA+ domain containing protein, genetically interacts with the mitochondrial protein export machinery

Nupur Sharma and Christof Osman

Supplementary material

Table S1. Yeast strains used in this study.

Strain Name	Strain description	Mating type	Genotype	Source
yCO272	Wild-type	a	*leu2-3,112 can1-100 ura3-1 his3-11,15*	W303
yCO273	Wild-type	alpha	*leu2-3,112 can1-100 ura3-1 his3-11,15*	W303
yCO520	*Δyme2*	a	*leu2-3,112 trp1-1 can1-100 ura3-1 ADE2-1 his3-11,15 Δyme2::NatNT2*	This study
yCO521	*Δmdm38*	a	*leu2-3,112 can1-100 ura3-1 his3-11,15 Δmdm38::hphNT1*	This study
yCO522	*Δyme2Δmdm38*	a	*leu2-3,112 can1-100 ura3-1 his3-11,15 Δmdm38::hphNT1 Δyme2::NatNT2*	This study
yCO536	*Δmba1*	alpha	*leu2-3,112 can1-100 ura3-1 his3-11,15 Δmba1::hphNT1*	This study
yCO537	*Δyme2Δmba1*	alpha	*leu2-3,112 can1-100 ura3-1 his3-11,15 Δmba1::hphNT1 Δyme2::NatNT2*	This study
yCO541	*Δoxa1*	alpha	*leu2-3,112 can1-100 ura3-1 his3-11,15 Δoxa1::hphNT1*	This study
yCO542	*Δyme2Δoxa1*	alpha	*leu2-3,112 can1-100 ura3-1 his3-11,15 Δyme2::NatNT2 Δoxa1::hphNT1*	This study
yNS041	*Δyme2* + pCO216_*YME2*	a	*leu2-3,112 trp1-1 can1-100 ura3-1 ADE2-1 his3-11,15 Δyme2::NatNT2* pRS316-*YME2-Ura3*	This study
yNS050	*Δyme2Δmdm38* + pCO216_*YME2*	a	*leu2-3,112 can1-100 ura3-1 his3-11,15 Δmdm38::hphNT1 Δyme2::NatNT2* pRS316-*YME2-Ura3*	This study

yNS053	*Δyme2Δmdm38* + pCO216_*YME2* + pCO033	a	*leu2-3,112 can1-100 ura3-1 his3-11,15 Δmdm38::hphNT1 Δyme2::NatNT2 pRS316-YME2-Ura3 pRS315-Leu2*	This study
yNS054	*Δyme2Δmdm38* + pCO216_*YME2* + pCO033_*YME2*	a	*leu2-3,112 can1-100 ura3-1 his3-11,15 Δmdm38::hphNT1 Δyme2::NatNT2 pRS316-YME2-Ura3 pRS315-Yme2-Leu2*	This study
yNS055	*Δyme2Δmdm38* + pCO216_*YME2* + pCO033_*YME2*^{K393A}	a	*leu2-3,112 can1-100 ura3-1 his3-11,15 Δmdm38::hphNT1 Δyme2::NatNT2 pRS316-Yme2-Ura3 pRS315-Yme2*^{K393A}*-Leu2*	This study
yNS056	*Δyme2Δmdm38* + pCO216_*YME2* + pCO033_*YME2*^{D522A}	a	*leu2-3,112 can1-100 ura3-1 his3-11,15 Δmdm38::hphNT1 Δyme2::NatNT2 pRS316-Yme2-Ura3 pRS315-Yme2*^{D522A}*-Leu2*	This study
yNS057	*Δyme2Δmdm38* + pCO216_*YME2* + pCO033_*YME2*$^{K393A/D522A}$	a	*leu2-3,112 can1-100 ura3-1 his3-11,15 Δmdm38::hphNT1 Δyme2::NatNT2 pRS316-Yme2-Ura3 pRS315-Yme2*$^{K393A/D522A}$*-Leu2*	This study
yNS158	*Δyme2Δmdm38* + pCO216_*YME2* + pCO033_*YME2*^{R523A}	a	*leu2-3,112 can1-100 ura3-1 his3-11,15 Δmdm38::hphNT1 Δyme2::NatNT2 pRS316-Yme2-Ura3 pRS315-Yme2*^{R523A}*-Leu2*	This study
yNS160	*Δyme2Δmdm38* + pCO216_*YME2* + pCO033_*YME2*^{R565A}	a	*leu2-3,112 can1-100 ura3-1 his3-11,15 Δmdm38::hphNT1 Δyme2::NatNT2 pRS316-Yme2-Ura3 pRS315-Yme2*^{R565A}*-Leu2*	This study
yNS068	*Δyme2 + Leu2::Yme2-TAP*	a	*leu2-3,112 trp1-1 can1-100 ura3-1 ADE2-1 his3-11,15 Δyme2::NatNT2 Leu2:: Yme2-TAP-Ura3*	This study
yNS071	*Δyme2 + Leu2::Yme2*^{K393A}*-TAP*	a	*leu2-3,112 trp1-1 can1-100 ura3-1 ADE2-1 his3-11,15 Δyme2::NatNT2 Leu2:: Yme2*^{K393A}*-TAP-Ura3*	This study
yNS072	*Δyme2 + Leu2::Yme2*^{D522A}*-TAP*	a	*leu2-3,112 trp1-1 can1-100 ura3-1 ADE2-1 his3-11,15 Δyme2::NatNT2 Leu2:: Yme2*^{D522A}*-TAP-Ura3*	This study
yNS074	*Δyme2 + Leu2::Yme2*$^{K393A/D522A}$*-TAP*	a	*leu2-3,112 trp1-1 can1-100 ura3-1 ADE2-1 his3-11,15 Δyme2::NatNT2 Leu2:: Yme2*$^{K393A/D522A}$*-TAP-Ura3*	This study

yNS046	*YME2-9Myc*	a	*leu2-3,112 can1-100 ura3-1 his3-11,15 Yme2-9xMyc-kanMX4*	This study
yNS171	*YME2-6HA*	alpha	*leu2-3,112 can1-100 ura3-1 his3-11,15 Yme2-6xHA-NatNT2*	This study
yNS172	*YME2-9Myc/YME2-6HA*	diploid	*leu2-3,112 can1-100 ura3-1 his3-11,15 Yme2-9xMyc-kanMX4/Yme2-6xHA-NatNT2*	This study
yNS173	*YME2-9Myc/YME2*	diploid	*leu2-3,112 can1-100 ura3-1 his3-11,15 Yme2-9xMyc-kanMX4/Yme2*	This study
yNS174	*YME2-6HA/YME2*	diploid	*leu2-3,112 can1-100 ura3-1 his3-11,15 Yme2-6xHA-NatNT2/Yme2*	This study
yNS129	*Δyme2Δmdm38* + pCO216_*YME2* + pCO033	a	*leu2-3,112 can1-100 ura3-1 his3-11,15 Δmdm38::hphNT1 Δyme2::NatNT2 pRS316-YME2-Ura3 pRS315-Leu2*	This study
yNS130	*Δyme2Δmdm38* + pCO216_*YME2*+ pCO033_*YME2*	a	*leu2-3,112 can1-100 ura3-1 his3-11,15 Δmdm38::hphNT1 Δyme2::NatNT2 pRS316-Yme2 pRS315-Yme2-Leu2*	This study
yNS131	*Δyme2Δmdm38* + pCO216_*YME2*+ pCO033_*YME2*^{F204A}	a	*leu2-3,112 can1-100 ura3-1 his3-11,15 Δmdm38::hphNT1 Δyme2::NatNT2 pRS316-Yme2 pRS315-Yme2*^{F204A}*-Leu2*	This study
yNS132	*Δyme2Δmdm38* + pCO216_*YME2*+ pCO033_*YME2*^{Y242A}	a	*leu2-3,112 can1-100 ura3-1 his3-11,15 Δmdm38::hphNT1 Δyme2::NatNT2 pRS316-Yme2 pRS315-Yme2*^{Y242A}*-Leu2*	This study
yNS133	*Δyme2Δmdm38* + pCO216_*YME2*+ pCO033_*YME2*$^{F204A/Y242A}$	a	*leu2-3,112 can1-100 ura3-1 his3-11,15 Δmdm38::hphNT1 Δyme2::NatNT2 pRS316-Yme2 pRS315-Yme2*$^{F204A/Y242A}$*-Leu2*	This study
yNS162	*Δyme2 + Leu2::Yme2*^{F204A}*-TAP*	a	*leu2-3,112 trp1-1 can1-100 ura3-1 ADE2-1 his3-11,15 Δyme2::NatNT2 Leu2:: Yme2*^{F204A}*-TAP-Ura3*	This study

yNS163	*Δyme2 + Leu2::Yme2*^{Y242A}*-TAP*	a	*leu2-3,112 trp1-1 can1-100 ura3-1 ADE2-1 his3-11,15 Δyme2::NatNT2 Leu2:: Yme2*^{Y242A}*-TAP-Ura3*	This study
yNS164	*Δyme2 + Leu2::Yme2*$^{F204A/Y242A}$*-TAP*	a	*leu2-3,112 trp1-1 can1-100 ura3-1 ADE2-1 his3-11,15 Δyme2::NatNT2 Leu2:: Yme2*$^{F204A/Y242A}$*-TAP-Ura3*	This study
yNS146	*Δyme2 Δmba1 + Leu2::Yme2-TAP*	a	*leu2-3,112 trp1-1 can1-100 ura3-1 ADE2-1 his3-11,15 Δyme2::NatNT2 Δmba1:: hphNT1 Leu2:: Yme2-TAP-Ura3*	This study
yNS150	*Δyme2 Δmdm38 + Leu2::Yme2-TAP*	a	*leu2-3,112 trp1-1 can1-100 ura3-1 ADE2-1 his3-11,15 Δyme2::NatNT2 Δmdm38:: hphNT1 Leu2:: Yme2-TAP-Ura3*	This study
yNS175	*Δyme2* + pCO033	a	*leu2-3,112 can1-100 ura3-1 his3-11,15 Δyme2::NatNT2 pRS315-Leu2*	This study
yNS176	*Δyme2* + pCO033_*YME2*	a	*leu2-3,112 can1-100 ura3-1 his3-11,15 Δyme2::NatNT2 pRS315-Yme2-Leu2*	This study
yNS177	*Δyme2* + pCO033_*YME2*^{K393A}	a	*leu2-3,112 can1-100 ura3-1 his3-11,15 Δyme2::NatNT2 pRS315-Yme2*^{K393A}*-Leu2*	This study
yNS178	*Δyme2* + pCO033_*YME2*^{D522A}	a	*leu2-3,112 can1-100 ura3-1 his3-11,15 Δyme2::NatNT2 pRS315-Yme2*^{D522A}*-Leu2*	This study
yNS179	*Δyme2* + pCO033_*YME2*$^{K393A/D522A}$	a	*leu2-3,112 can1-100 ura3-1 his3-11,15 Δyme2::NatNT2 pRS315-Yme2*$^{K393A/D522A}$*-Leu2*	This study
yNS180	*Δyme2* + pCO033_*YME2*^{F204A}	a	*leu2-3,112 can1-100 ura3-1 his3-11,15 Δyme2::NatNT2 pRS315-Yme2*^{F204A}*-Leu2*	This study
yNS181	*Δyme2* + pCO033_*YME2*^{Y242A}	a	*leu2-3,112 can1-100 ura3-1 his3-11,15 Δyme2::NatNT2 pRS315-Yme2*^{Y242A}*-Leu2*	This study
yNS182	*Δyme2* + pCO033_*YME2*$^{F204A/Y242A}$	a	*leu2-3,112 can1-100 ura3-1 his3-11,15 Δyme2::NatNT2 pRS315-Yme2*$^{F204A/Y242A}$*-Leu2*	This study
yNS183	*Δyme2* + pCO033_*YME2*^{R523A}	a	*leu2-3,112 can1-100 ura3-1 his3-11,15 Δyme2::NatNT2 pRS315-Yme2*^{R523A}*-Leu2*	This study

yNS185	*Δyme2* + pCO033_*YME2*^{R565A}	a	*leu2-3,112 can1-100 ura3-1 his3-11,15 Δyme2::NatNT2 pRS315-Yme2*^{R565A}*-Leu2*	This study

Table S2. Plasmids used in this study.

Plasmid name	Description	Resistance/ Auxotrophy marker	Source
pCO021	pFA6a	*kanMX4*	Janke et al. 2004
pCO059	pFA6a	*NatNT2*	Janke et al. 2004
pCO074	pFA6a	*hphNT1*	Janke et al. 2004
pCO214	pYM13-TAP	*kanMX4*	Janke et al. 2004
pCO340	pYM17-6HA	*NatNT2*	Janke et al. 2004
pCO341	pYM18-9Myc	*kanMX4*	Janke et al. 2004
pCO216	pRS316-Yme2	*Ura3*	This study
pCO033	pRS315	*Leu2*	This study
pNS019	pRS315-*Yme2*	*Leu2*	This study
pNS020	pRS315-Yme2^{K393A}	*Leu2*	This study
pNS022	pRS315-Yme2^{D522A}	*Leu2*	This study
pNS024	pRS315-Yme2$^{K393A/D522A}$	*Leu2*	This study
pNS049	pRS315-Yme2^{F204A}	*Leu2*	This study
pNS051	pRS315-Yme2^{Y242A}	*Leu2*	This study
pNS053	pRS315-Yme2$^{F204A/Y242A}$	*Leu2*	This study
pNS065	pRS315-Yme2^{R523A}	*Leu2*	This study
pNS067	pRS315-Yme2^{R565A}	*Leu2*	This study
pNS026	*Leu2::YME2*-TAP	*Ura3*	This study
pNS029	*Leu2::Yme2*^{K393A}-TAP	*Ura3*	This study
pNS030	*Leu2::Yme2*^{D522A}-TAP	*Ura3*	This study
pNS032	*Leu2::Yme2*$^{K393A/D522A}$-TAP	*Ura3*	This study
pNS069	*Leu2::Yme2*^{F204A}-TAP	*Ura3*	This study
pNS070	*Leu2::Yme2*^{Y242A}-TAP	*Ura3*	This study
pNS071	*Leu2::Yme2*$^{F204A/Y242A}$ -TAP	*Ura3*	This study

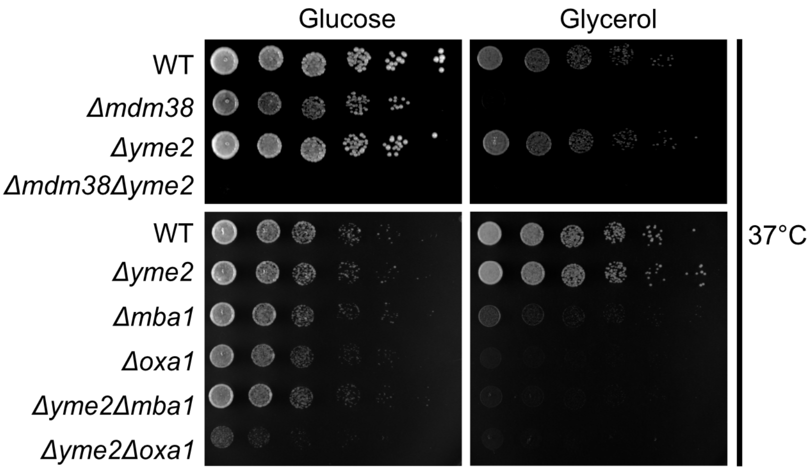

Figure S1 Genetic interactions of YME2 with components of the protein biogenesis machinery at 37°C. Growth test analysis showing the genetic interactions of YME2 with MDM38, MBA1 and MBA1. The indicated strains were grown to logarithmic phase and spotted on fermentable glucose medium and non-fermentable glycerol medium and incubated at 37°C for 1 day (in case of growth of Δmba1, Δoxa1, Δyme2Δmba1, Δyme2Δoxa1 on glycerol) and 2 days (in case of the remaining strains).

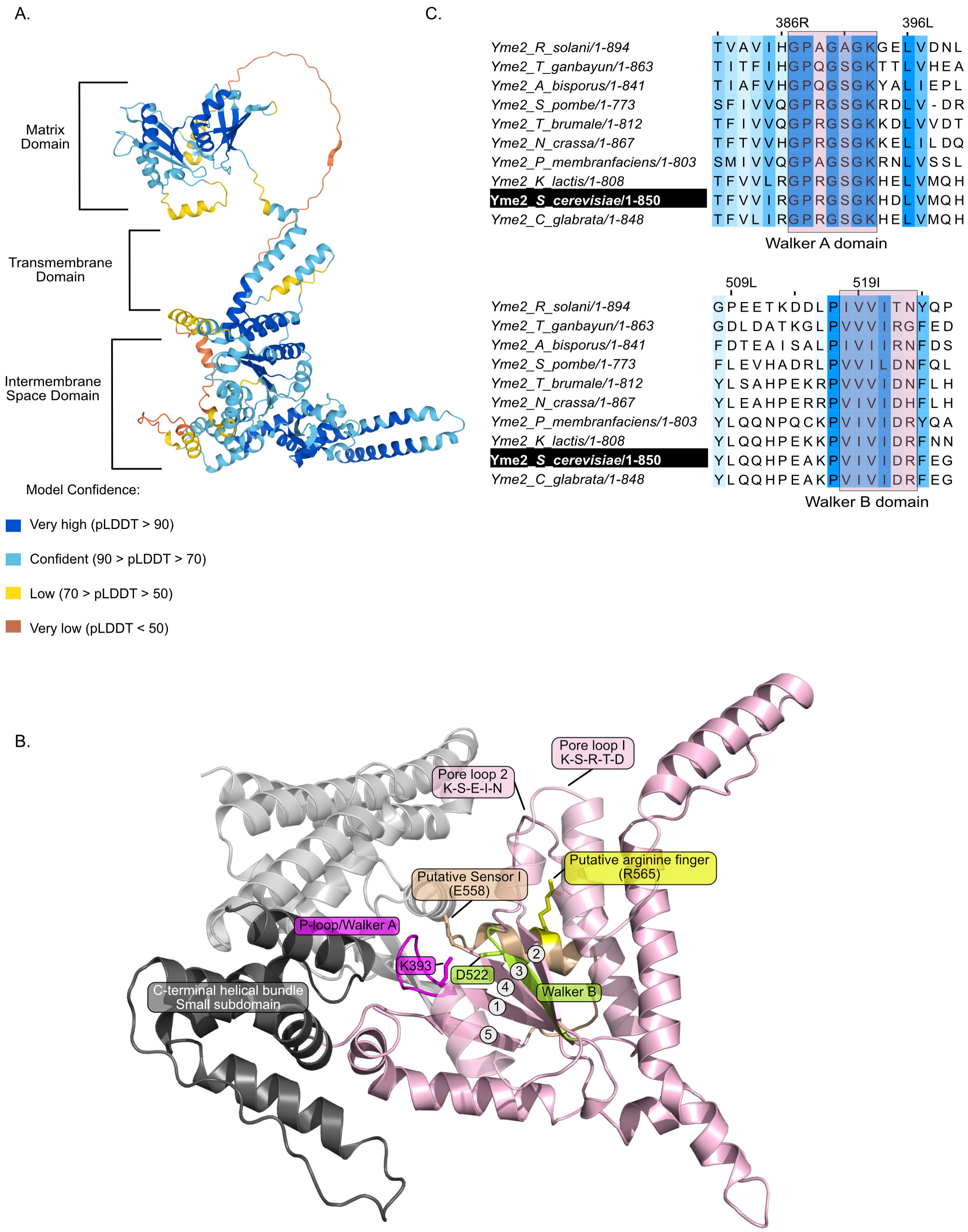

A.
Matrix Domain
Transmembrane Domain
Intermembrane Space Domain
Model Confidence:
Very high (pLDDT > 90)
Confident (90 > pLDDT > 70)
Low (70 > pLDDT > 50)
Very low (pLDDT < 50)
C.
386R
396L
Yme2_R_solani/1-894 TVAVIHGPAGAGKGELVDNL
Yme2_T_ganbayun/1-863 TITFIHGPQGSGKTTLVHEA
Yme2_A_bisporus/1-841 TIAFVHGPQGSGKYALIEPL
Yme2_S_pombe/1-773 SFIVVQGPRGSGKRDLV-DR
Yme2_T_brumale/1-812 TFIVVQGPRGSGKKDLVVDT
Yme2_N_crassa/1-867 TFTVVHGPRGSGKKELILDQ
Yme2_P_membranfaciens/1-803 SMIVVQGPAGSGKRNLVSSL
Yme2_K_lactis/1-808 TFVVLRGPRGSGKHELVMQH
Yme2_S_cerevisiae/1-850 TFVVIRGPRGSGKHDLVMQH
Yme2_C_glabrata/1-848 TFVLIRGPRGSGKHELVMQH
Walker A domain
509L
519I
Yme2_R_solani/1-894 GPEETKDDLPIVVITNYQP
Yme2_T_ganbayun/1-863 GDLDATKGLPVVVIRGFED
Yme2_A_bisporus/1-841 FDTEAISALPIVIIRNFDS
Yme2_S_pombe/1-773 FLEVHADRLPVVILDNFQL
Yme2_T_brumale/1-812 YLSAHPEKRPVVVIDNFLH
Yme2_N_crassa/1-867 YLEAHPERRPVIVIDHFLH
Yme2_P_membranfaciens/1-803 YLQQNPQCKPVIVIDRYQA
Yme2_K_lactis/1-808 YLQQHPEKKPVIVIDRFNN
Yme2_S_cerevisiae/1-850 YLQQHPEAKPVIVIDRFEG
Yme2_C_glabrata/1-848 YLQQHPEAKPVIVIDRFEG
Walker B domain
B.
Pore loop I
K-S-R-T-D
Pore loop 2
K-S-E-I-N
Putative arginine finger
(R565)
Putative Sensor I
(E558)
P-loop/Walker A
K393
D522
Walker B
C-terminal helical bundle
Small subdomain
1
2
3
4
5

D.

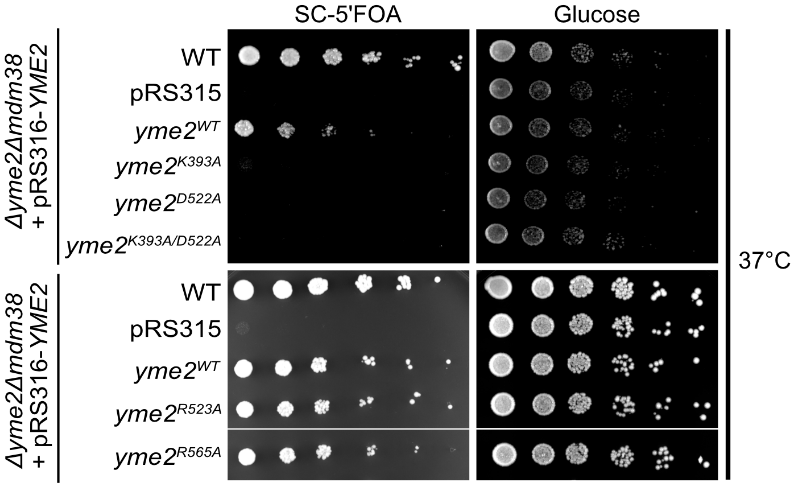

E.

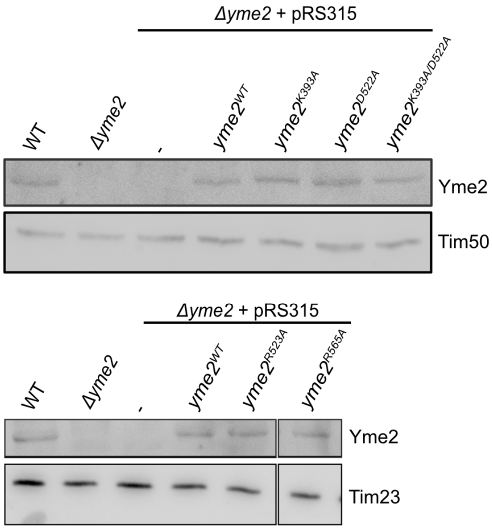

Figure S2 Predicted structural model of the AAA+ domain of Yme2 and its functional analysis (A) Predicted structural model for Yme2 in S.cerevisiae. The model was predicted using AlphaFold (https://alphafold.ebi.ac.uk/). The putative domains are as indicated. The color scheme represents the per residue confidence score, or pLDDT, as indicated in the schematic. (B) Structural prediction of the AAA+ fold of Yme2 using AlphaFold, indicating the Walker A and B motifs, the putative Arginine finger, the Pore loops, the Sensor I motif and the C-terminal helical bundle. The numbers indicate the β-sheets. (C) A schematic showing multiple sequence alignment of Yme2 across 10 fungal species generated with the Jalview program from the MUSCLE alignment tool (https://www.ebi.ac.uk/Tools/msa/muscle/). The alignment shows the conservation of putative Walker A and B motifs of the AAA+ domain with the Yme2 sequence of S.cerevisiae set as reference. The blue color intensity depicts the conservation of each residue, and the respective motifs are highlighted in red. (D) Growth test analysis showing the plasmid shuffle experiment performed with the mutants from the AAA+ domain at 37°C. The indicated strains were grown to logarithmic phase and the serial dilutions were spotted on fermentable glucose medium (YPD) and on SC+ 5'FOA and incubated for 2 days. (E) Western blot showing the expression levels of the pRS315 plasmids with the indicated mutants transformed into a Δyme2 strain. The blot was decorated with Yme2 antibody and Tim23 antibody was used as a loading control.

A.

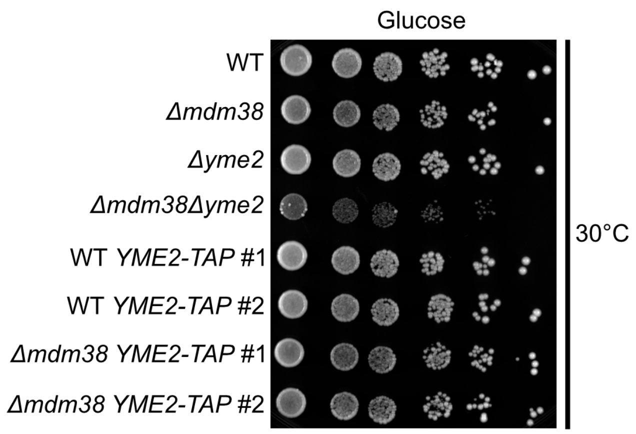

B.

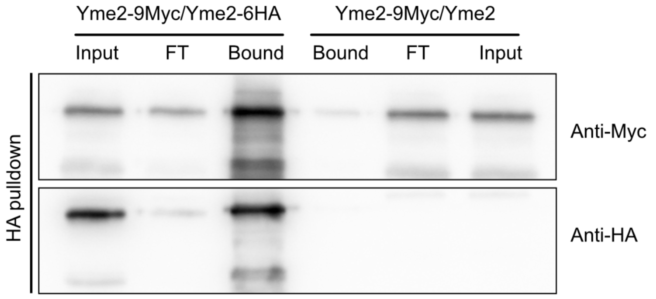

Figure S3 Immunoprecipitation experiments depicting the presence of multiple copies of Yme2 in the complex (A) Growth test analysis depicting the TAP-tagged YME2 strain in WT and Δmdm38 background. The indicated strains were grown to logarithmic phase before spotting on fermentable glucose medium and incubated at 30°C for 2 days. (B) Western blot showing the HA-immunoprecipitation experiment performed with the indicated diploid strains. For each strain, 1% of the input and flowthrough (FT), and 50% of the bound fractions were loaded. The blot was decorated with Anti-HA and Anti-Myc antibody.

A.

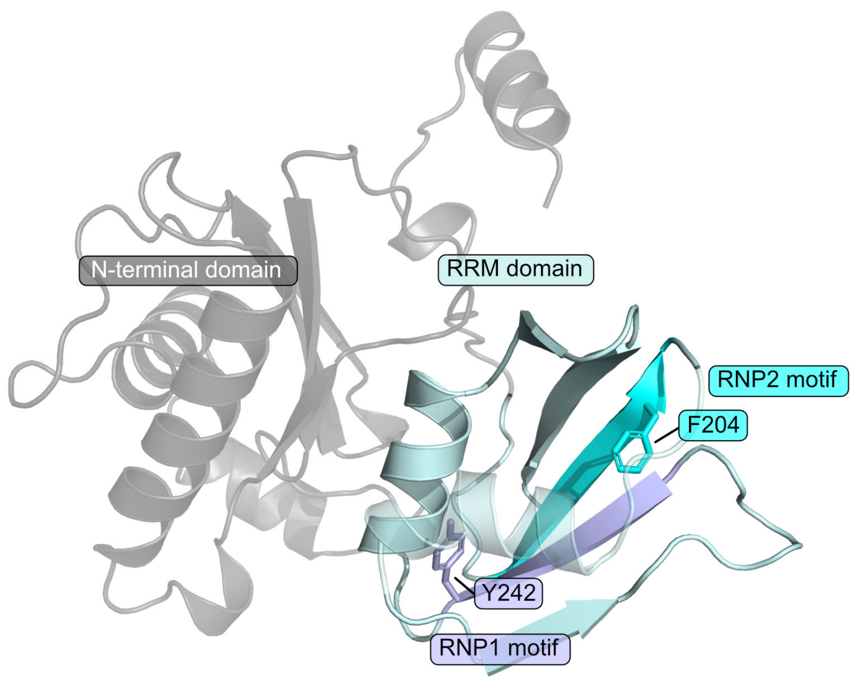
N-terminal domain
RRM domain
RNP2 motif
F204
Y242
RNP1 motif

B.

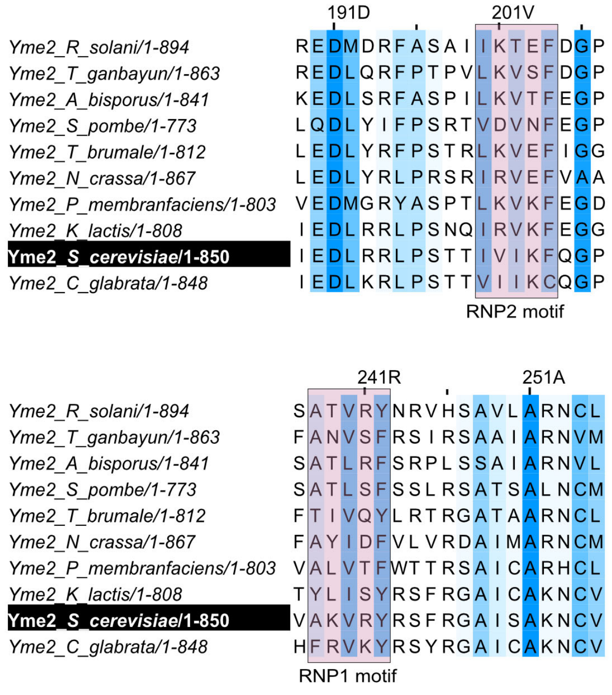
191D
201V
Yme2_R_solani/1-894 REDMDRFASAIIKTEFDGP
Yme2_T_ganbayun/1-863 REDLQRFPTPVLKVSFDGP
Yme2_A_bisporus/1-841 KEDLSRFASPILKVTFEGP
Yme2_S_pombe/1-773 LQDLYIFPSRTVDVNFEGP
Yme2_T_brumale/1-812 LEDLYRFPSTRLKVEFIGG
Yme2_N_crassa/1-867 LEDLYRLPRSRIRVEFVAA
Yme2_P_membranfaciens/1-803 VEDMGRYASPTLKVKFEGD
Yme2_K_lactis/1-808 IEDLRRLPSNQIRVKFEGG
Yme2_S_cerevisiae/1-850 IEDLRRLPSTTIVIKFQGP
Yme2_C_glabrata/1-848 IEDLKRLPSTTVIIKCQGP
RNP2 motif
241R
251A
Yme2_R_solani/1-894 SATVRYNRVHSAVLARNCL
Yme2_T_ganbayun/1-863 FANVSFRSIRSAAIARNVM
Yme2_A_bisporus/1-841 SATLRFSRPLSSAIARNVL
Yme2_S_pombe/1-773 SATLSFSSLRSATSALNCM
Yme2_T_brumale/1-812 FTIVQYLRTRGATAARNCL
Yme2_N_crassa/1-867 FAYIDFVLVRDAIMARNCM
Yme2_P_membranfaciens/1-803 VALVTFWTTRSAICARHCL
Yme2_K_lactis/1-808 TYLISYRSFRGAICAKNCV
Yme2_S_cerevisiae/1-850 VAKVRYRSFRGAISAKNCV
Yme2_C_glabrata/1-848 HFRVKYRSYRGAICAKNCV
RNP1 motif

C.

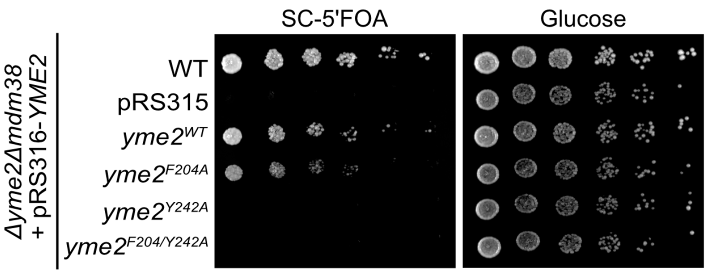

D.

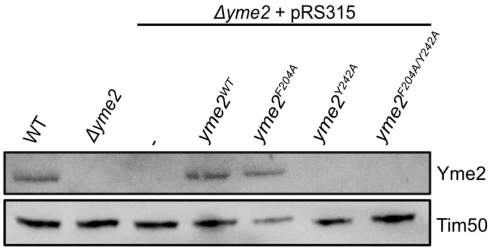

Figure S4 Predicted structural model of the RRM domain of Yme2 and its functional analysis (A) Structural prediction of the RRM domain of Yme2, indicating the RNP2 and RNP1 motifs, along with the residues that were mutated in this study. The colors indicate the different motifs, blue-RNP2 motif; purple- RNP1 motif. (B) A schematic showing multiple sequence alignment of Yme2 across 10 fungal species generated with the Jalview program from the MUSCLE alignment tool (https://www.ebi.ac.uk/Tools/msa/muscle/). The alignment shows the conservation of putative RNP motifs of the RRM domain with the Yme2 sequence of S.cerevisiae set as reference. The blue color intensity depicts the conservation of each residue, and the respective motifs are highlighted in red. (C) Growth test analysis showing the plasmid shuffle experiment performed with the mutants from the RRM domain at 37°C. The indicated strains were grown to logarithmic phase and the serial dilutions were spotted on fermentable glucose medium (YPD) and on SC+ 5’FOA and incubated for 2 days. (D) Western blot showing the expression levels of the pRS315 plasmids with the indicated mutants transformed into a Δyme2 strain. The blot was decorated with Yme2 antibody and Tim50 antibody was used as a loading control.
